# Estimation of Muscle Mass in the Integrated Assessment of Patients on Hemodialysis

**DOI:** 10.3389/fnut.2021.697523

**Published:** 2021-08-16

**Authors:** Alice Sabatino, Natascha J. H. Broers, Frank M. van der Sande, Marc H. Hemmelder, Enrico Fiaccadori, Jeroen P. Kooman

**Affiliations:** ^1^Nephrology Unit, Department of Medicine and Surgery, Parma University Hospital, University of Parma, Parma, Italy; ^2^Division on Nephrology, Department of Internal Medicine, Maastricht University Medical Centre, Maastricht, Netherlands; ^3^NUTRIM School of Nutrition and Translational Research in Metabolism, CARIM School for Cardiovascular Diseases, Maastricht University, Maastricht, Netherlands

**Keywords:** ultrasound, bioimpedance, sarcopenia, hemodialysis, creatinine index

## Abstract

Assessment of muscle mass (MM) or its proxies, lean tissue mass (LTM) or fat-free mass (FFM), is an integral part of the diagnosis of protein-energy wasting (PEW) and sarcopenia in patients on hemodialysis (HD). Both sarcopenia and PEW are related to a loss of functionality and also increased morbidity and mortality in this patient population. However, loss of MM is a part of a wider spectrum, including inflammation and fluid overload. As both sarcopenia and PEW are amendable to treatment, estimation of MM regularly is therefore of major clinical relevance. Whereas, computer-assisted tomography (CT) or dual-energy X-ray absorptiometry (DXA) is considered a reference method, it is unsuitable as a method for routine clinical monitoring. In this review, different bedside methods to estimate MM or its proxies in patients on HD will be discussed, with emphasis on biochemical methods, simplified creatinine index (SCI), bioimpedance spectroscopy (BIS), and muscle ultrasound (US). Body composition parameters of all methods are related to the outcome and appear relevant in clinical practice. The US is the only parameter by which muscle dimensions are measured. BIS and SCI are also dependent on either theoretical assumptions or the use of population-specific regression equations. Potential caveats of the methods are that SCI can be influenced by residual renal function, BIS can be influenced by fluid overload, although the latter may be circumvented by the use of a three-compartment model, and that muscle US reflects regional and not whole body MM. In conclusion, both SCI and BIS as well as muscle US are all valuable methods that can be applied for bedside nutritional assessment in patients on HD and appear suitable for routine follow-up. The choice for either method depends on local preferences. However, estimation of MM or its proxies should always be part of a multidimensional assessment of the patient followed by a personalized treatment strategy.

## Introduction

Assessment of nutritional state is of high relevance in patients on hemodialysis (HD). This given the relation between protein-energy wasting (PEW), a condition characterized by reduced body stores of protein and energy fuels characteristic of patients with chronic kidney disease (CKD) and end-stage kidney disease (ESKD), and mortality (1), and since abnormalities in the nutritional state may be amenable to therapeutic intervention (2). Muscle wasting in patients on HD can be due to multiple factors including insufficient dietary intake and a loss of nutrients through the dialysate, or an increased muscle breakdown due to inflammation or metabolic acidosis (3). Measurement of muscle mass (MM), or its proxies lean tissue mass (LTM) or fat-free mass (FFM), is an integral part of the assessment of the nutritional state, as well as in the diagnosis of sarcopenia. Sarcopenia is characterized by reduced MM and strength and is frequently observed in the elderly, but can also happen earlier as a consequence of chronic conditions, such as CKD/ESKD and patients on HD (3–5). Malnutrition as well as sarcopenia are part of a spectrum including impaired functional status, low physical activity, low quality of life, and frailty (6–8), and they are the important components of the premature aging phenomenon in this patient population (9). Next to this, inflammation and fluid overload were also found to be related to a decrease in LTM and intracellular water (ICW) (8, 10). Thus, loss of MM is a central part of the multimorbid spectrum of patients on HD, and should be interpreted in view of both its consequences as well as in the context of potentially amendable underlying factors. The aim of this short review is to give a concise overview of instrumental methods that can be used on a daily clinical basis in patients on HD, and their use in the context of a multidimensional assessment in these patients will be discussed.

## Clinical Syndromes Associated With a Loss of MM

Loss of MM or its proxies is included in various syndromes related to the nutritional and functional status of the patient on HD, as summarized in Table 1. Except frailty, in which only a reduction in muscle strength is a parameter, a reduction of MM or FFM is included in the diagnostic criteria of other syndromes, such as PEW, cachexia, and sarcopenia. These partly, but not entirely, overlapping syndromes (3, 11) are part of a wide spectrum of nutritional and functional abnormalities in patients on HD, although an important common denominator appears to be tissue loss (11). Importantly, one of the criteria for the definition of PEW, also referred to as kidney cachexia, is an increase in inflammatory parameters (4, 12). In contrast, inflammation is not included in the diagnostic criteria of sarcopenia (13). This division is relevant, as the pathophysiology and also possibly the clinical approach to a patient on HD with a pure “sarcopenic” phenotype may differ from that of a patient with a “cachectic” phenotype (14). Furthermore, patients can have both an increase in fat mass and a decline in MM (15), an entity also known as sarcopenic obesity, which is prevalent in patients on HD, although its relation with the outcome is yet uncertain (16, 17). The development of sarcopenic obesity is not captured by the estimation of changes in body weight or body mass index (BMI) (18).

**Table 1:** Categories of assessment for the diagnosis of different syndromes related to the nutritional and functional status of patients.

**Criteria**	**Malnutrition ESPEN**	**Malnutrition GLIM**	**PEW**	**Cachexia**	**Sarcopenia**	**Frailty**
Weight loss/BMI	+	+	+	+		+
Muscle mass/FFM/LTM	+	+	+	+	+	
Muscle strength					+	+
Functional performance					+	+
Fatigue/exhaustion						+
Biochemical markers			+	+		
Etiology		+				

*BMI, body mass index; FFM, fat free mass; LTM, lean tissue mass; PEW, protein energy wasting*.

**Figure 1:**
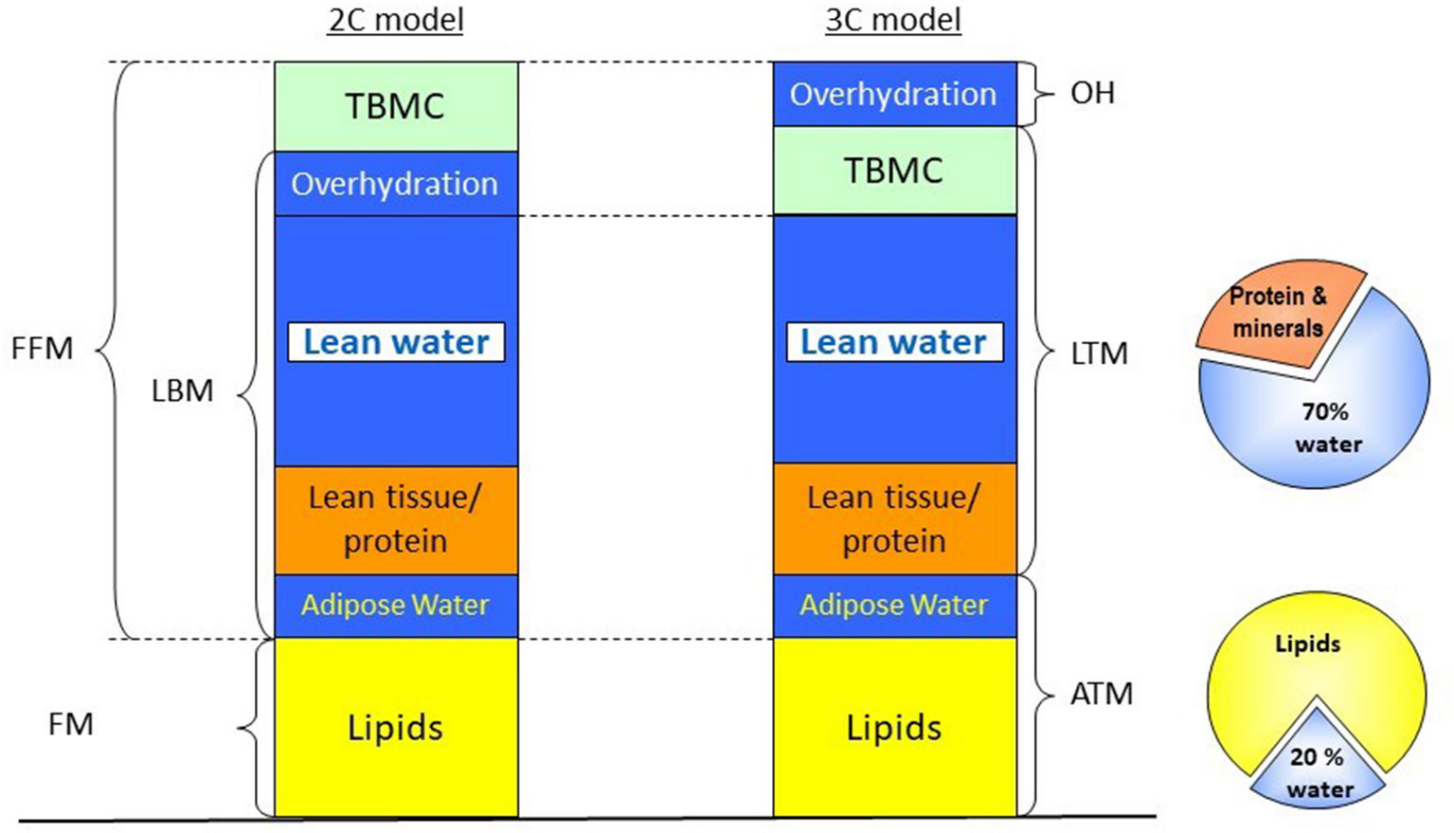
Different body compartment models based on BIA, BIS, and DXA. In the BIA approach, a two-compartment model is applied, dividing the body in FFM, which includes bone mineral tissue, total body water, skeletal muscle and visceral proteins, and FM. Both DXA and three-compartment BIS apply a three-compartment approach; however, in the case of DXA, bone mineral density is removed from the FFM, and LBM is measured instead, while in the case of BIS, a virtual “overhydration” compartment is calculated as the difference between measured and expected ECW, providing information on ATM and a normohydrate LTM, which includes bone mineral tissue. ATM, adipose tissue mass; BIA, bioelectrical impedance analysis; BIS, bioelectrical impedance spectroscopy; DXA, dual-energy X-ray absorptiometry; FFM, fat-free mass; FM, fat mass; LBM, lean body mass; LTM, lean tissue mass; OH, overhydration; TBMC, total body mineral content. Modified from Broers et al. (22) with permission.

The assessment of body composition is complicated by the fact that various parameters are used to express (loss) of MM or LBM. For instance, LBM, FFM, and MM are not equivalent, although they are often used as interchangeable surrogates. FFM, as the name suggests, is the total body mass except for the body fat, and it includes LBM and bone mineral tissue. The LBM, in turn, is composed of the total body water, appendicular skeletal muscle mass (ASMM), and the fat-free mass of organs. Since different techniques measure different compartments, the identification of the body compartment of interest, along with the availability of the method, must precede the choice of the method of assessment. As an example, some available techniques, such as bioelectrical impedance analysis (BIA), assess body composition by dividing the body into two compartments (2-C), the FFM, which conceptually includes all non-fat tissue and the fat mass (FM) (19). While other methods, such as dual-energy X-ray absorptiometry (DXA) and one application of bioelectrical impedance spectroscopy (BIS), divides the body into three compartments (3C). DXA assesses LBM, which includes total body protein and total body water (TBW), but excludes bone and fat mass (20, 21) (Figure 1). On the other hand, 3C-BIS assess a “normohydrated” LTM, which reflects a compartment that is separate from adipose tissue mass (ATM; fat mass and adipose water) and a virtual “overhydration” compartment, but that includes bone mineral tissue (8) (Figure 1), as will be discussed later in more detail.

These specific examples show that although all entities reflect comparable physiological dimensions, they are not interchangeable. FFM assessed by a 2-C model can be different from LTM assessed by a 3-C model (22). Thus, parameters obtained by a specific method cannot be used interchangeably with comparable parameters assessed by different methodologies. In addition, ideally, reference values should also be developed for specific techniques, devices, and populations. As this may not always be feasible, it is important to have these caveats in mind when assessing literature on body composition in patients on kidney replacement therapy (KRT).

## Bedside Technology For the Assessment of MM

For the assessment of MM or its proxies, various options are available. Computer-assisted tomography (CT) or MRI are considered gold standard methods but are impractical to be used on a routine basis (13, 23). DXA is generally considered a reference method to estimate LBM as well as ASMM in guidelines (13) but may be difficult to perform frequently in clinical practice. Furthermore, because DXA assumes a hydration ratio with LBM of 0.73 (24), results can be influenced by severe fluid overload (25). Still, DXA, when routinely available, provides important information on changes in body composition on HD and might also serve as a calibration for bedside methods.

Various methods are available to assess MM or LBM in patients which can be used on a routine basis in patients on KRT. These can be conceptually divided into methods that indirectly estimate body composition (such as bioimpedance or anthropometry), biochemical methods (based on creatinine kinetics), and methods that measure muscle dimensions at an anatomical level [MRI, CT, or ultrasound (US)]. Anthropometry is a time-honored method that is also included in the original diagnostic criteria for PEW (4). When performed by a skilled investigator it was able to predict a reduction in MM with an accuracy comparable with instrumental methods (16). However, the emphasis of the present article is on biochemical and technological tools in the assessment of body composition.

### Bioelectrical Impedance

Although the theoretical backgrounds of BIA are complex and discussed in excellent reviews (26, 27), in general, this method measures the opposition (impedance) of the body or a body segment to an alternating current. The impedance (*Z*) is a composite of the resistance to this flow, which is related to TBW, and reactance (Xc) is related to the capacitance of the cellular membrane. With the single-frequency (SF) approach, FFM and ASMM are estimated using population-derived regression equations, including Z or R, measured at 50 kHz as the resistance index H^2^/R50 along with anthropometric parameters, sex, and age (27). With SF-BIA, also ASMM can be estimated using regression equations (27, 28).

The multifrequency (MF) approach delivers different frequencies that vary from 5 to 1,000 kHz, and depending on the method it can use only several frequencies or, in the case of BIS, broadband of frequencies within this range. The lower the frequency, the more the difficulty with which it has to pass across the cell, passing only at the extracellular water (ECW) at frequencies < than 1 kHz and not through the ICW, with higher frequencies it passes through both, with TBW being measured at frequencies > 5,000 kHz. For technical reasons, measurements at very low and very high frequencies are not possible; however, with BIS, the resistance at zero (Ro) and infinity (R ∞) are extrapolated by applying a Cole-Cole plot to predict ECW and TBW (27). In classic 2C-models of SF-BIA and MF-BIA, FFM is subsequently calculated from TBW, assuming fractional hydration of 0.73 (29). A drawback of this method, which divides the body into two compartments, is that the excess of ECW due to fluid overload is added to the TBW which can subsequently result in an overestimation of FFM (25) (Figure 1). In the case of BIS, Moissl et al. developed a model for the assessment of ECW, ICW, and TBW using Hanai mixture theory adjusted for BMI, which showed good agreement with dilution methods (30). Chamney et al. further developed the so-called 3C-model, which assumes fixed hydration of LTM and ATM, and divides the body in normohydrate LTM and ATM, and a virtual “overhydration” (OH) compartment (31).

With regard to the estimation of body composition, a definite superiority of either approach has not yet been proven. Donadio et al. found a slightly lower prediction error for FFM with SF-BIA as compared with an MF-BIA using a two-compartment approach, with a highly significant correlation of both methods with DXA (32). Another study observed a stronger relation between FFM estimated by MF-BIA and creatinine kinetics as compared with SF-BIA (33). Raimann et al. found a slightly improved estimation of ICW with the SF method, and conversely, improved estimation of ECW with BIS. With regard to the detection of changes in ECW, MF-BIA was found to have a higher precision (34, 35). On the other hand, ASMM predicted by the Sergi equation using SF-BIA showed high accuracy in predicting sarcopenia with DXA as the reference method (16); however, this equation with the form: ASMM (kg) = −3.964 + (0.227^*^[height (cm)]^2^/R]) + (0.095^*^weight) + (1.384^*^sex) + (0.064^*^Xc), was primarily validated in an elderly Caucasian population (36).

With the 3C BIS method, estimation of body composition is based on a theoretical approach without the use of population-specific regression equations. Lean tissue index (LTI), which corresponds to LTM, divided by height^2^, below the 10th percentile of a healthy age-matched reference population was independently related to outcome in most, but not all studies (8, 15, 37, 38). Still, in a meta-analysis including over 15,000 patients, a low LTI was associated with increased mortality [Hazard ratio 1.53 (95% CI: 1.41–1.64)] (39). Especially, the combination of a low LTI and fat tissue index (FTI, the height^2^-normalized ATM) appears to be associated with increased mortality risk (15).

However, in several studies, despite reasonable agreement at a population level, relatively wide limits of agreement were observed between body compartments assessed by 3C-BIS and reference techniques, such as DXA (40, 41). To some degree, these differences may be explained by the fact that even the reference method is not free of errors. Indeed, the excess ECW with overhydration is added to the LBM compartment with DXA, but not with the 3C-BIS approach (8). Also, it should be taken into account that ATM assessed by 3C-BIS includes intra adipose water, unlike FM measured by DXA. Using ASMM measured by DXA as the reference method, LTM measured by whole-body BIS was able to predict sarcopenia with acceptable accuracy (mean AUC 0.79 for females and 0.77 for males) (16); however, it should be noted that in the current EWGSOP2 guidelines, cut-off values based on the 3C-BIS model were not yet included in the definition, while the Sergi equation based on SF BIA was advocated for standardization (13). However, in the case of tissue edema, these estimations may be less reliable in patients on HD. To standardize measurements and avoid this kind of problem, the recent KDOQI guidelines on nutrition in CKD recommends that BIA/BIS should be performed at least 30 min after the HD session to allow for the distribution of body fluids (42). Still, in the case of 3C-BIS, measurements of ATM and normohydrated LTM were slightly (0.77 and 0.40 kg, respectively) affected by the timing of measurements (43), whereas predialytic fluid status was more consistently related to the outcome as compared with postdialytic measurements (44).

An alternative approach is the construction of a vector plotting R and Xc at 50 kHz within tolerance ellipses of a healthy population. The advantage of this method is that results are displayed without the need for underlying theoretical assumptions or population-based equations (28). A potential disadvantage is that the direct translation of the findings into constructs such as sarcopenia, fluid overload, and PEW may be more difficult as compared with a numerical approach.

To summarize, whereas various BIA approaches can be used to assess body composition in dialysis patients, it must be kept in mind that estimations are dependent on population-specific regression equations or theoretical assumptions regarding the conversion from bioelectrical signals to estimations of body water compartments. Measurements obtained with a specific device or method can therefore not be used interchangeably (45), even with regard to raw parameters such as Z, Xc, and R (46). It is important to acknowledge that as long as a device is correctly calibrated, the magnitude of the differences are small and generally within the precision of the specifications of the manufacturer. Whereas, a definite superiority of a specific BIA methodology for estimations of body composition has not been proven, in our view, the 3-C model holds the advantage that it also provides a separate estimation of fluid status in a single measurement, whereas it has shown high-construct validity in predicting outcome in large datasets.

### Biochemical Methods: The Creatinine Index

Serum creatinine is a breakdown product of creatine phosphate in muscle tissue that was found to be strongly related to LBM assessed by DXA in patients on HD (47). Serum creatinine is inversely related to mortality in patients on renal replacement therapy (48); however, serum creatinine in patients on HD is also dependent on dialysis adequacy. Therefore, the concept of creatinine index (CI) was developed (49). CI was found to be an independent predictor of outcome in patients with HD (50). However, as creatinine kinetics may be complicated to use in routine clinical practice, a simplified form [simplified creatinine index (SCI)] was developed, which was also found to be related to the outcome (51). SCI (mg/kg/d) is calculated according to the formula 16.21 + 1.12^*^ [1 if male; 0 if female]−0.06^*^age (years) −0.08^*^spKt/V urea + 0.009^*^predialytic serum creatinine (μmol/l). Also, LTI derived from SCI was strongly related to LTI assessed by BIS, although the mean BIS-derived value was 4.7 kg lower than the SCI-estimated value (51). SCI is easy to apply in clinical practice as only routinely gathered data that are already present in electronic health records (EHR) are needed, with the advantage that longitudinal trends can be tracked easily. Indeed, SCI declined 6 months before death, potentially serving as an early warning sign (51). However, a potential pitfall in the follow-up of the SCI is changes in residual renal function, which may independently affect serum creatinine values apart from muscle mass, as well as changes in dietary intake (42). Serum creatinine is also a parameter included in the nutritional component score (NCS), an aggregate score that also consists of routinely captured parameters interdialytic weight gain, serum phosphate, serum albumin, and normalized protein catabolic rate (nPCR). The use of this score, which was shown to decline 1–2 months before hospitalization and also up to 6 months before death (52, 53), allows for the interpretation of changes in parameters like interdialytic weight gain (IDWG) and serum phosphate, which have a bidirectional relationship with the outcome (54). Whereas, a decrease in these parameters is usually regarded as a positive sign given their detrimental effect on the cardiovascular status of the patient, a sharp decline in IDWG and serum phosphate can also be a sign of impending malnutrition and adverse outcomes when accompanied by a decrease in the other nutritional parameters (52).

To Summarize, following trends in biochemical indices derived from EHR can aid in the early detection of changes in nutritional state and MM, whereas changes in serum creatinine or SCI cannot replace validated questionnaires to establish the risk for sarcopenia (55), they can aid in case finding given the fact that they can be performed on a frequent and routine basis with the potential for automated processing of the findings.

### Muscle Ultrasound

Recently, regional muscle US has been applied in patients with kidney disease for the assessment and monitoring of skeletal muscle. Its major advantages, compared to other imaging techniques, are represented by lower cost, portability, lack of radiation exposure, and the possibility to be applied by non-specialized staff (56–58). In comparison to other bedside techniques, such as anthropometry, US allows real-time visualization of the target structure, allowing for the assessment of muscle size (thickness and area) and/or quality, through echogenicity, which provides information about the presence of inflammation, fibrosis, and adipose infiltration (59). Its portability is of particular interest in the CKD research setting and clinical practice since patients can be evaluated during HD session or outpatient visits.

**Figure 2:**
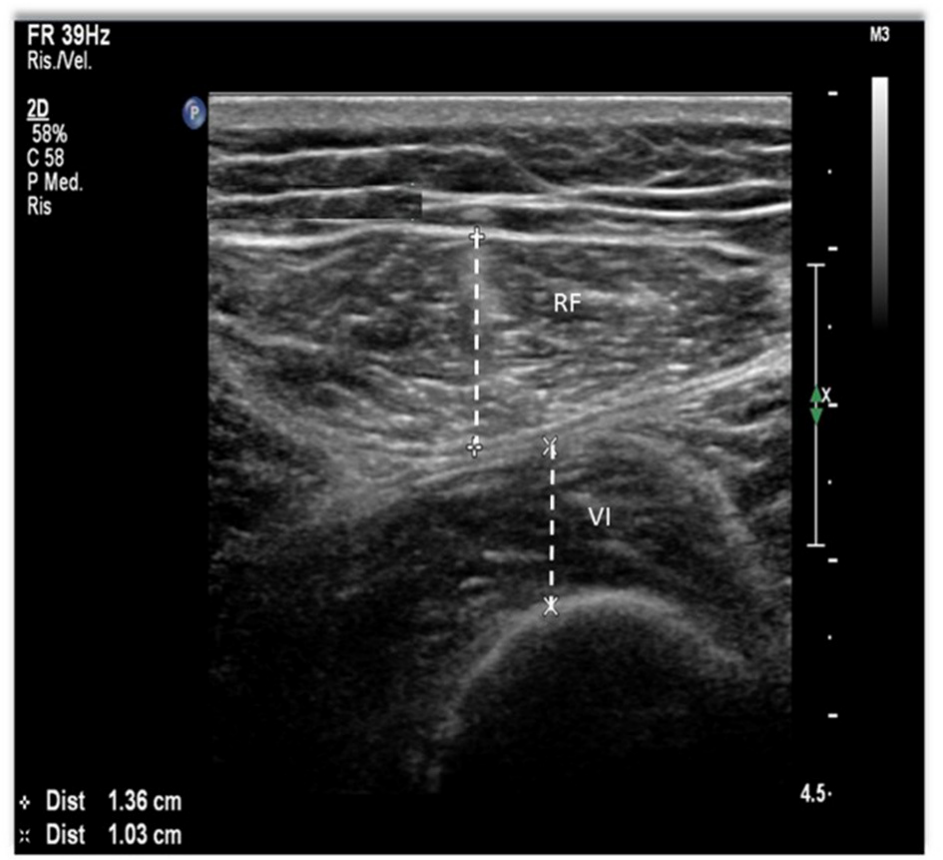
Quadriceps muscle ultrasound (US) methodology. The points of interest correspond to the midpoint and the lower third between the anterior superior iliac spine (ASIS) and the upper pole of the patella. Using a B-mode ultrasound with a linear transducer, we obtain the image on the right. Rectus femoris (RF) and vastus intermedius (VI) thickness, a measure on the inner edge of the muscle.

Quadriceps muscle US has been studied extensively in patients with renal disease. In the available research, two muscles were most frequently studied, the quadriceps rectus femoris (RF) and vastus intermedius (VI), in two different points, the midpoint, and at the border of the lower third and upper two-thirds between the anterior superior iliac spine and the upper pole of the patella (Figure 2) (56, 57, 60–62). Abundant contact gel to avoid any pressure is needed to prevent muscle deformation. Regarding the accuracy and reproducibility of the method, its reliability has been tested in critically ill patients with acute kidney injury (AKI), showing excellent intraclass correlation coefficients (ICC) for inter- and intra-operator comparisons, as well as for measurements performed before and after HD (56). In the same clinical setting, US assessment of quadriceps muscle has also been validated against CT (60), showing small and non-significant differential and proportional bias in comparison with CT (60). Also in patients with AKI, US was successfully used to monitor the quadriceps muscle in the first 5 days of stay in the intensive care unit, being able to identify early muscle loss (63). In non-acutely ill patients, US was performed in patients on HD before and after the dialysis session to assess whether the presence of fluid overload or the rapid fluid shifts caused by the treatment could influence measurements (57). No differences were found between measurements performed before and after dialysis, and the correlation between measurements was very high, ranging from 0.91 to 0.95 (57), showing that muscle US is not influenced by fluid overload. Also in the outpatient setting, the assessment of muscle cross-sectional area (CSA) was validated using CT in patients with CKD and not on HD (61). In another study, RF-CSA was assessed before and after 12 weeks of resistance exercise in patients with CKD not on HD, showing a high correlation with MRI at baseline and follow-up, and moderate positive association observed between changes in RF-CSA by US and quadriceps volume by MRI following exercise training (64).

More recently, studies investigating the role of quadriceps muscle US in identifying patients with PEW or sarcopenia have been published. Sabatino et al. (57) used the PEW cutoffs for BMI and albumin to stratify patients on chronic HD in two groups and found that in the multivariable analysis patients with lower BMI had lower muscle thickness, whereas no difference was found between patients with serum albumin below or above the reference value (57). A similar analysis was performed using the malnutrition inflammation score (MIS), an internationally recognized tool to assess malnutrition and predict outcomes in patients on HD, and found that patients with worse scores (≥ 6) had lower RF and VI thickness (57). In another study, quadriceps CSA cut-offs have been derived using receiver-operation characteristic curves based on the presence or absence of PEW in a Malaysian population (62). In that study, patients diagnosed with PEW had significantly lower RF and VI thickness and RF CSA in comparison with well-nourished patients, and the area under the curve (AUC) for RF CSA was high (men = 0.74, 95% CI: 0.66–0.82 and women = 0.82, 95% CI: 0.73–0.91, both *p* < 0.001). In addition, the correlation between US and LTI by BIS ranged from moderate to high (0.28–0.52) depending on the measurement site, with a higher correlation for RF thickness and CSA in comparison to VI.

Despite such encouraging results, more work is needed before assuming muscle US as a reference method for the diagnosis of sarcopenia. Studies defining reference values derived from healthy subjects from populations with different ethnic backgrounds should be performed to allow the early identification of patients with low MM. However, considering its validity, reliability, and sensitivity in detecting changes in skeletal muscle, its use as a tool for the monitoring of the regional muscularity of a patient could be recommended.

### The Role of Estimation of MM in the Integrated Functional Assessment of Patients on KRT

Assessment of MM or its derivatives achieves its full potential in combination with other parameters. As shown in Table 1, with a relatively limited battery of measurements, various clinical syndromes such as PEW, sarcopenia, and frailty can be easily diagnosed. For the diagnosis of sarcopenia, it should be combined with an assessment of muscle strength, for instance, handgrip strength (HGS), and in case of a positive diagnosis, with a measure of physical function such as the 4 m gait speed test (13). HGS and the gait speed tests are easy to perform even in a routine clinical setting. Muscle quantity and strength, though interrelated (22), are not equivalent. Muscle strength appears to be a more powerful predictor for the outcome as compared to MM (65). Furthermore, following renal transplantation, we observed a profound increase in HGS without a significant increase in LTM (66).

Which bedside test should be used for the assessment of MM/FFM/LBM depends on local preferences and availability. In the case of 3C-BIS, information on body composition as well as the fluid overload is combined in a single measurement. Muscle ultrasound may be superior to BIS in the diagnosis of skeletal muscle depletion, but reference values need to be defined in larger populations, whereas a trained investigator is necessary. A possibility is to use SCI or BIS for case finding, followed by the US for a more precise estimate of MM depletion.

**Figure 3:**
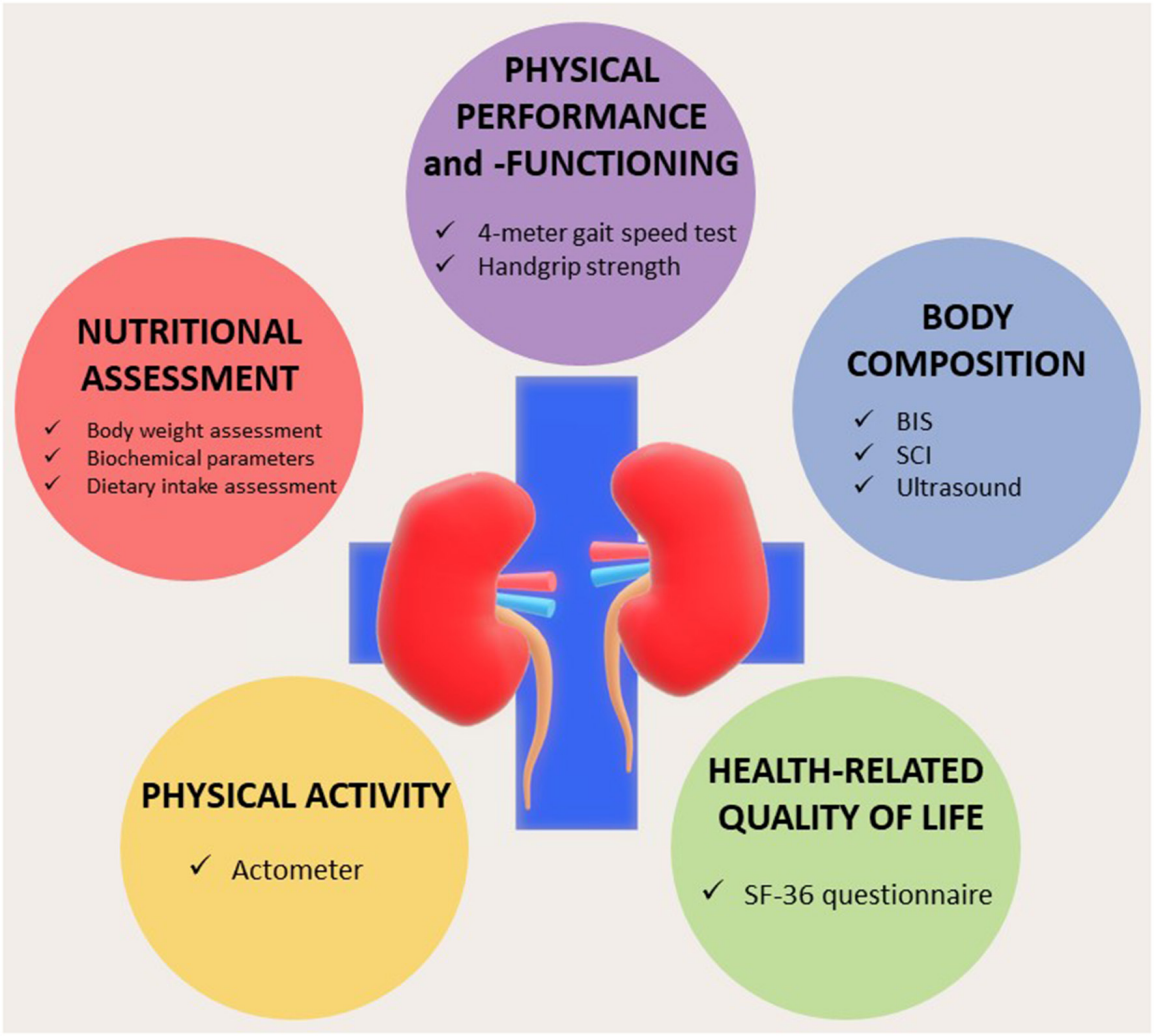
Dashboard summarizing the integrated nutritional assessment of patients with chronic kidney disease (CKD)/end-stage kidney disease (ESKD). BIS, bioelectrical impedance spectroscopy; SCI, simplified creatinine index. Modified from Broers et al. (8) under Creative Commons.

A diagnosis of PEW or sarcopenia should be combined with an estimate of potentially modifiable factors such as dietary intake and physical activity, for example, by performing actimetry regularly. Also, impaired physical activity, nutritional status, and physical performance should be interpreted given its relation with a low-health-related QoL (7, 67), which is especially important as these factors are often amenable to therapeutic intervention. Lastly, complications that are frequently associated with loss of MM, such as inflammation and fluid overload, should be assessed. A proposal for an integrated assessment, preferably summarized in an easily interpretable dashboard, is illustrated in Figure 3. Such a dashboard could be the basis for a personalized approach. For instance, a patient with a low LTI or MM and reduced muscle strength with adequate nutritional intake and absence of inflammation (a “sarcopenic” phenotype), but with low physical activity may primarily benefit from both aerobic as well as resistance training (68). In addition, activity trackers or smartphone applications may provide the patient with feedback on his/her physical activity patterns.

On the other side of the spectrum, a patient with a “cachectic” phenotype, with inflammation, and reduced protein intake, will primarily benefit from a search into the cause of inflammation and targeted nutritional intervention. Participation in an active rehabilitation program will be much more difficult for this patient, although interventions such as neuromuscular electrical stimulation may be beneficial (69). In addition, the risk of fluid overload may be increased in this patient, which may only be resolved by an increase in dialysis time due to the altered distribution between the interstitial and intravascular compartment (70).

In conclusion, a reduction in MM is an important determinant of various clinical syndromes in patients on dialysis, which are related to increased morbidity and mortality but also potentially amenable to therapeutic intervention. Whereas, different bedside methods can be used to assess MM or its proxies in patients on dialysis, it is important to maintain a critical view of their relative advantages and potential pitfalls. Assessment of MM should be part of a multidimensional approach and a personalized treatment strategy.

## Author Contributions

AS and JK conceived the paper. NB, FS, MH, and EF contributed to and reviewed the paper. All authors contributed to the article and approved the submitted version.

## Conflict of Interest

The authors declare that the research was conducted in the absence of any commercial or financial relationships that could be construed as a potential conflict of interest.

## Publisher's Note

All claims expressed in this article are solely those of the authors and do not necessarily represent those of their affiliated organizations, or those of the publisher, the editors and the reviewers. Any product that may be evaluated in this article, or claim that may be made by its manufacturer, is not guaranteed or endorsed by the publisher.
